# A predictive model of muscle excitations based on muscle modularity for a large repertoire of human locomotion conditions

**DOI:** 10.3389/fncom.2015.00114

**Published:** 2015-09-17

**Authors:** Jose Gonzalez-Vargas, Massimo Sartori, Strahinja Dosen, Diego Torricelli, Jose L. Pons, Dario Farina

**Affiliations:** ^1^Neural Rehabilitation Group, Cajal Institute, Spanish National Research CouncilMadrid, Spain; ^2^Department of Neurorehabilitation Engineering, University Medical Center GöttingenGöttingen, Germany

**Keywords:** muscle synergies, human locomotion, muscle modularity, Human modeling, neuromusculoskeletal system

## Abstract

Humans can efficiently walk across a large variety of terrains and locomotion conditions with little or no mental effort. It has been hypothesized that the nervous system simplifies neuromuscular control by using muscle synergies, thus organizing multi-muscle activity into a small number of coordinative co-activation modules. In the present study we investigated how muscle modularity is structured across a large repertoire of locomotion conditions including five different speeds and five different ground elevations. For this we have used the non-negative matrix factorization technique in order to explain EMG experimental data with a low-dimensional set of four motor components. In this context each motor components is composed of a non-negative factor and the associated muscle weightings. Furthermore, we have investigated if the proposed descriptive analysis of muscle modularity could be translated into a predictive model that could: (1) Estimate how motor components modulate across locomotion speeds and ground elevations. This implies not only estimating the non-negative factors temporal characteristics, but also the associated muscle weighting variations. (2) Estimate how the resulting muscle excitations modulate across novel locomotion conditions and subjects. The results showed three major distinctive features of muscle modularity: (1) the number of motor components was preserved across all locomotion conditions, (2) the non-negative factors were consistent in shape and timing across all locomotion conditions, and (3) the muscle weightings were modulated as distinctive functions of locomotion speed and ground elevation. Results also showed that the developed predictive model was able to reproduce well the muscle modularity of un-modeled data, i.e., novel subjects and conditions. Muscle weightings were reconstructed with a cross-correlation factor greater than 70% and a root mean square error less than 0.10. Furthermore, the generated muscle excitations matched well the experimental excitation with a cross-correlation factor greater than 85% and a root mean square error less than 0.09. The ability of synthetizing the neuromuscular mechanisms underlying human locomotion across a variety of locomotion conditions will enable solutions in the field of neurorehabilitation technologies and control of bipedal artificial systems. Open-access of the model implementation is provided for further analysis at https://simtk.org/home/p-mep/.

## Introduction

Human locomotion emerges from the complex interaction between the nervous, muscular, and skeletal systems (Winter, 1990; Enoka, 2008; Latash, 2010). Human body is a multiple degree of freedom (DOF) structure, comprising many segments connected by joints, actuated by a highly redundant set of non-linear actuators (muscles). In spite of this complexity humans can walk efficiently across a variety of (irregular) terrains and they can seamlessly transit across locomotion speeds and ground elevations, while adapting to the underlying mechanical demand with little or no mental effort (Clark, 2015). The ability to understand how the neuromuscular controller handles this complexity and dimensionality is fundamental to understand, characterize, and synthetize human movement.

Bernstein (1967) hypothesized that the nervous system simplifies the motor control by organizing the high-dimensional neuromuscular activity into a small number of coordinative modules. Based on this, later studies introduced the concept of muscle synergies (d'Avella et al., 2003; Bizzi et al., 2008; Overduin et al., 2012). Muscle synergies are considered to be the basic control signals responsible for generating the larger repertoire of muscle-specific excitation needed for executing a specific motor task (d'Avella et al., 2003; Cheung et al., 2005; Ting and Macpherson, 2005; Bizzi et al., 2008; Dominici et al., 2011; Bizzi and Cheung, 2013). These hypotheses have been supported by a number of experimental studies conducted in animal models (d'Avella et al., 2003; Ting and Macpherson, 2005; Overduin et al., 2008) as well as in the intact human (Lacquaniti et al., 2012; Lafortune et al., 2012; Duysens et al., 2013) and in patients with neurological impairments (Gizzi et al., 2011). In these studies, muscles electromyograms (EMGs) were recorded experimentally and used to explore neuromuscular control strategies in a descriptive manner. Typically, collected EMG signals are linearly separated into various motor components by using techniques such as non-negative matrix factorization (NNMF) (Lee and Seung, 2001) or principal/independent component analysis (Cappellini and Ivanenko, 2006). In the case of the NNMF each motor component is composed of a non-negative factor and the associated muscle weightings. In the context of this manuscript NNMF is the factorization technique employed to carry out the descriptive analysis of muscle modularity. Whether this approach can also be used to predict (rather than describe) patterns of muscle excitation is an open question, which we aim to address in this manuscript.

In this study we addressed three major questions. Firstly, we investigated experimentally how muscle modularity is structured across a large repertoire of human locomotion tasks, i.e., five locomotion speeds and five ground elevations, for a total of 25 conditions. Secondly, we extracted regularities characterizing the experimentally observed muscle modularity. Regularities were used to build a predictive model that could be employed to generate motor components as well as muscle-specific excitation patterns required to walk at a given speed and elevation. Finally, we validated the ability of the predictive model to yield electrophysiologically consistent estimates of muscle excitations over non-modeled locomotion conditions and subjects. In summary, the relevance of this study include:

1. Understanding whether muscle modularity preserves its structure over a large repertoire of locomotion conditions (e.g., including for the first time various combinations of speed and elevation conditions). This is an open question in current movement neurophysiology and biomechanics.
2. Understanding whether it is possible to reliably synthetize the neuromuscular control signals underlying human locomotion into a compact computational model. This model would provide biologically inspired controllers to be employed in neuromusculoskeletal simulations and neurorehabilitation technologies.

## Materials and methods

### Experimental procedures

Nine healthy male subjects of age: 31.1 ± 5.5 years, weight: 73.7 ± 10.51 kg, and height: 1.76 ± 0.08 m (mean ± standard deviation) volunteered for the experiments. The Ethics Committee of the University Medical Center Goettingen approved the experimental protocol. All participants signed an informed consent. Subjects walked on a treadmill across 25 locomotion conditions including five speeds (i.e., 1, 2, 3, 4, and 5 km/h) and five elevations (i.e., −20, −10, 0, 10, and 20%). Subjects performed on average 17 ± 2.5 gait cycles per condition measured starting from the heel strike.

During treadmill locomotion, EMG signals were band-pass filtered between 10 and 500 Hz and recorded at the sampling frequency of 2048 Hz using a multi-channel amplifier (USB-EMG2, OTBioelettronica, IT) connected to disposable Ag-AgCl electrodes (Neuroline 720, Ambu, USA) in bipolar configuration. Data were recorded from 15 muscle groups of the right leg including: Tibialis Anterior (TA), Soleus (Sol), Peroneus (Per), Vastus Lateralis (VastLat), Vastus Medialis (VastMed), Rectus Femoris (RFem), Sartorius (Sar), Adductor Group (Add), Gluteus Medius (GlutMed), Tensor Fasciae Latae (TFL), Gastrocnemius Lateralis (GastLat), Gastrocnemius Medialis (GastMed), Biceps Femoris (BFem), Semimembranosus (Sem), and Gluteus Maximus (GlutMax). The electrodes were placed following the SENIAM^1^ recommendations and using manual palpation to cross check. Before placing the electrodes, the skin was prepared by shaving the target area and by applying a small quantity of abrasive gel (Everi, Spes Medica, IT). A retro-reflective marker was placed on the heel of the right leg and recorded using a seven-camera motion capture system (Oqus cameras, Qualysis, SE).

### Movement data processing

The raw heel marker trajectories obtained from the motion tracking system were smoothed using a low-pass (8 Hz), zero-phase, fourth-order Butterworth filter. This marker trajectory was used to detect the initial contact of the foot with the ground and to segment the gait cycle. Acquired EMG data were band-pass filtered (30–300 Hz) using a zero-phase, fourth-order Butterworth filter to remove movement artifacts, full-wave rectified and then low-pass filtered (3 Hz) using the same filter type to determine linear envelopes. For each subject and muscle group, the resulting linear envelopes were normalized with respect to the overall peak amplitude for that muscle. This was determined as the maximum value of a 50 ms moving-average window applied to the muscle linear envelopes across all the recorded trials. Normalized linear envelopes were then segmented into individual gait cycles based on the heel marker trajectories. The amplitude-normalized EMG linear envelopes were then time-normalized to 200 equidistant points over a gait cycle using a cubic spline function, and the resulting profiles were referred to as the experimental muscle excitations.

### Descriptive analysis

This analysis was performed using data of seven subjects across all (25) conditions. For every subject and locomotion condition, the muscle excitations from each gait cycle were concatenated into an *m* × *n* matrix, where *m* indicates the number of muscles and *n* is the number of time-normalized samples (i.e., 200) multiplied by the number of gait cycles. The NNMF algorithm was applied to the experimental muscle excitation matrix resulting in a set of subject-specific and condition-specific motor components, i.e., a non-negative factor and associated discrete weightings (Lee and Seung, 2001). The number of motor components was an input parameter for the NNMF algorithm and it constrained the final factorization dimensionality. The extracted non-negative factors were linearly combined with the weightings to produce an *m* × *n* matrix of reconstructed excitations, which was then compared to the experimental muscle excitation matrix. The accuracy of the reconstruction was assessed with the Variance Accounted For (VAF) index, defined as *VAF* = 1 – *SSE*/*TSS*, where *SSE* is the sum of squared errors between the experimental and reconstructed excitations and represents the unexplained variation, and *TSS* is the total sum of squares, which quantifies the total variation of the experimental excitations (Ivanenko et al., 2006a; Dominici et al., 2011; Sartori et al., 2013). For a given number of motor components, this procedure was iterated 50 times starting from randomly chosen initial conditions for the factors and weightings (Ivanenko et al., 2005; Gizzi et al., 2011; Sartori et al., 2013). Across all 50 factorizations, the one with the highest VAF was considered as the final output for that specific dimensionality. The dimensionality was incrementally increased until a minimal threshold VAF of 85% was obtained, and the resulting set of motor components was deemed to be the final output of the factorization (Gizzi et al., 2011; Lacquaniti et al., 2012; Sartori et al., 2013).

Within each motor component, the resulting non-negative factors were averaged across all gait cycles and normalized with respect to their maximum value. The associated muscle weightings were then scaled accordingly, i.e., by the inverse of the normalization coefficient. Therefore, averaged and normalized non-negative factors varied between 0 and 1 and encoded the temporal modulation of muscle recruitment. On the other hand, the scaled weightings encoded the amplitude information (Sartori et al., 2013).

### Predictive model

**Figure 1:**
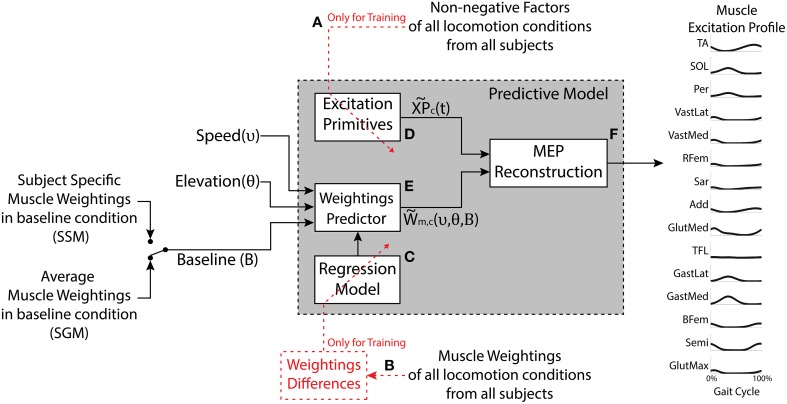
**The model has three inputs: The desired speed, the desired elevation, and the set of muscle weightings characterizing the baseline condition (elevation 0% and speed 3 km/h)**. The outputs are predicted motor components (weightings and excitation primitives) as well as the synthetic muscle excitation profiles (MEP) for the muscles under study. The generic Excitation Primitive block **(D)** was determined using the non-negative factors collected experimentally **(A)**. The Regression Model block **(C)** was determined using experimental muscle weightings **(B)**. The Weightings Predictor block **(E)** produces the estimated muscle weightings for a given speed and elevation. The MEP Reconstruction block **(F)** realizes the linear combination of the excitation primitives and the estimated muscle weightings. The model can be applied in two modes, the Subject-Generic Mode (SGM) and the Subject-Specific Mode (SSM), depending whether the input baseline weightings are generic (subject group average) or experimentally obtained for a specific subject.

Figure 1 shows a block diagram of the developed predictive model. The model estimates: (1) non-negative factors, (2) muscle weightings, and (3) the resulting muscle excitation profiles (MEP). These were estimated for a given locomotion condition (i.e., speed and elevation) and a set of weightings characterizing the baseline condition (elevation 0% and speed of 3 km/h). In summary, the model was constructed based on the findings from the descriptive analysis, which demonstrated that the structure of the locomotion motor program, including the number and timing of the non-negative factors, was consistent across the conditions. Therefore, subject-specific and condition-specific profiles were averaged and parameterized to determine a set of generic factors (Figure 1A). The muscle weightings, on the other hand, modulated systematically across the conditions and these trends were captured using regression with the elevation and speed as the independent variables (Figure 1B). The regression was determined with respect to the differences in the weightings relative to their absolute values in the baseline condition.

The development of the predictive model is explained thoroughly in the Supplementary Material and it comprises three major blocks:

- **Excitation primitives block (Figure** **1D****)**. This block approximates the experimental non-negative factors for each motor component (*c*) using single impulse Gaussian curves similarly to what has been reported in literature (Ivanenko et al., 2006a; Gizzi et al., 2011; Duysens et al., 2013; Sartori et al., 2013). These curves explain the temporal modulation of the non-negative factor profiles observed in the descriptive analysis as a function of the percentage of the gait cycle. We refer to this single impulse Gaussian curves as the “excitation primitives,” or XPs (Sartori et al., 2013):
  (1) $${\tilde{XP}}_{c}\left(t\right)=e^{-\frac{{\left(t-\mu_{c}\right)}^{2}}{2\sigma_{c}{}^{2}}}$$
  where *t* is the gait cycle frame (i.e., 0% ≤ *t* ≤ 100% gait cycle), μ_*c*_ is the temporal shift of the peak of the Gaussian curve within the gait cycle, and σ_*c*_ is the width of the Gaussian curve for each extracted component (*c*). Refer to Section Experimental Procedures of the Supplementary Material.
- **The weightings predictor block (Figure 1E)**. This block takes as input locomotion speed (υ) and elevation (θ) and computes the resulting weightings for a selected muscle (m) and motor component (c). The model employs the following regression equation:
  (2) $$W_{m,c}\left(\upsilon ,\theta ,W_{BL}\right)=\Delta \upsilon {\left(\upsilon \right)}_{m,c}+\Delta \theta {\left(\theta \right)}_{m,c}+W_{BL_{m,c}}$$
  where *W*_*BL*_ are the muscle weightings at the baseline locomotion condition, and Δυ and Δθ are the increments modeling the additive changes in the baseline weightings due to the velocity and elevation, respectively. Refer to Section Movement Data Processing and Descriptive Analysis of the Supplementary Material for more detail.
- **The MEP reconstruction block (Figure 1F)**. This block multiplies the XPs $\left({\tilde{XP}}_{c}\left(t\right)\right)$ from the first block (Figure 1D) by the muscle weightings $\left({\tilde{W}}_{m,c}\left(\upsilon ,\theta ,W_{BL}\right)\right)$ estimated in the second block (Figure 1F) to predict the excitation profile $\left({\tilde{MEP}}_{m}(\upsilon ,\theta ,W_{BL})\right)$ of a specific muscle (*m*) at a desired velocity (υ) and elevation (θ), as given in Equation (3) (refer to Section Predictive Model of the Supplementary Material):
  (3) $${\tilde{MEP}}_{m}(\upsilon ,\theta ,W_{BL})={\tilde{W}}_{m,c}(\upsilon ,\theta ,W_{BL})\cdot {\tilde{XP}}_{c}\left(t\right)$$

#### Subject-generic or subject-specific modes

The predictive model can output subject-specific or subject-generic MEPs depending on the set of baseline weightings used to calibrate it (as described by Equation 2). The subject-generic estimation was determined by averaging the weightings in the baseline condition per muscle and component across all the subjects in the training dataset (i.e., mean experimental baseline). When such a baseline is used as an input for the predictive model (Figure 1), the resulting estimations reflect an average motor control across a group of subjects. The calibration using the average baseline allows the model to be applied as is, without the need to collect additional experimental data. On the other hand, for the subject-specific estimation, the model is calibrated using the baseline weightings obtained experimentally from an individual subject walking at baseline condition. In this, we hypothesize that the model estimations for other conditions would be in this case more precise since the model is customized to an individual subject. Two uses of the model are hereafter denoted as a subject-generic mode (SGM) and subject-specific mode (SSM), respectively.

## Analysis and validation procedures

### Training and validation scenarios

**Figure 2:**
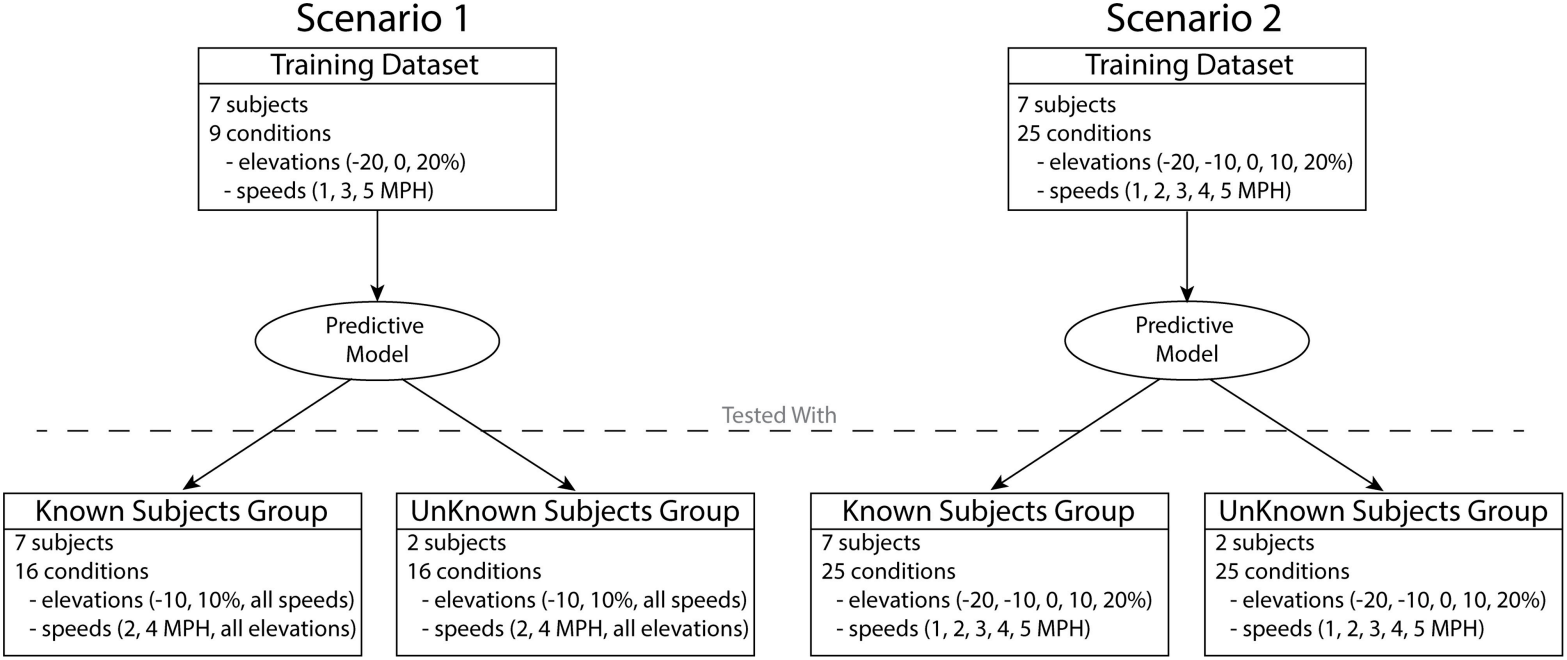
**Two different scenarios were used in order to test the predictive model**. The model in scenario 1 was developed using the data from seven subjects over nine conditions. The model in scenario 2 was developed using the data from seven subjects in full set of 25 conditions. The models developed in each scenario were assessed using the data of the seven subjects included in the training (known subjects group) as well as the two subjects excluded from the training (unknown subjects group).

Two different scenarios were used in order to develop and test the predictive model (Figure 2). In both scenarios, the data obtained by the descriptive analysis of seven subjects were used to train the predictive model, i.e., to determine the XPs and regression equations. The two remaining subjects were used to further test both scenarios with novel subjects (unknown subject group). In scenario 1, the training included only the data from a subset of conditions, three elevations (−20, 0, and 20%) and three speeds (1, 3, and 5 km/h), for a total of nine conditions. This scenario yielded a predictive model trained on a reduced dataset. This enabled assessing the ability of a conservative model to generalize predictions of experimental data in novel locomotion conditions and subjects. Therefore, the model was tested with the remaining 16 conditions of the seven subjects (known subject group) as well as with the two unknown subjects excluded from the training (unknown subject group).

In scenario 2 the training step included all 25 locomotion conditions, thus showing the capacity of a more complete model to predict known and unknown subject groups. The predictive model trained and validated under this scenario will be freely released to the public upon acceptance.

It is worth stressing that scenario 1 represents this study's main validation procedure. Scenario 2 allowed validating the comprehensive predictive model that is finally released to the public and therefore provides the perspective user with expected predictive performance analyses.

### Descriptive analysis

The extracted non-negative factors and muscle weightings were compared across subjects, locomotion conditions, and gait cycles. The time shift between non-negative factors was computed as the time difference required to align the signals for maximum correlation and expressed as a percentage of the gait cycle. Then, the cross-correlation coefficient (*r*) was calculated after compensating for this time shift. These measures assessed the average similarity in the factor shapes and timing across the conditions. Statistically significant differences for the weighting amplitudes of each muscle across the conditions were tested using a Two-Way repeated measures ANOVA with speed and elevation as the factors. The significance threshold was set at *p* < 0.05 using the Greenhouse-Geisser correction.

### Predictive model

The predictive model performance was evaluated following the two scenarios described in Section Training and Validation Scenarios (Figure 2). In each scenario, the model was applied in both modes (SSM and SGM), and the assessment considered the outputs of each model block:

- **Evaluation of the XP block output**. Cross-correlation coefficient (*r*) and the time shift were computed to compare the similarity between the experimental non-negative factors and generic XPs across subjects and conditions.
- **Evaluation of the weightings predictor block output**. To assess an overall similarity between the sets of predicted and experimental muscle weightings, the cross correlation coefficient (*r*) was determined by treating a set of weightings within a single motor component as a vector of values. In addition, the root mean square error (*RMSE*) between the estimated and experimentally obtained values was computed for each weighting within each component to assess an average absolute error in estimating a specific weighting. Since, there was a large set of test cases, histograms were used to report concisely the individual results and demonstrate the overall performance of the model. Furthermore, due to the non-normality of the data, the median and the interquartile range were used to report the overall performance. Also, the Wilcoxon Signed Ranks test was used to compare the quality of estimation when using SGM vs. SSM. The threshold was set at *p* < 0.05.
- **Evaluation of the MEP predictor block output**. The resulting MEPs obtained using the predictive model in SSM and SGM were compared with the experimental muscle excitations by computing *r* and the *RMSE* between the predicted and experimental profiles. Histograms, median and interquartile range were used to report concisely the overall performance of the model. Also, the Wilcoxon Signed Ranks test was applied to assess the differences in performance between the SGM and the SSM.

## Results

### Descriptive analysis

**Figure 3:**
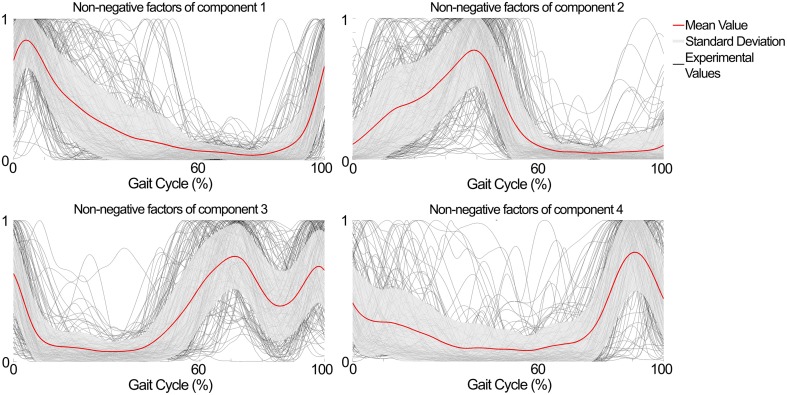
**Subject- and condition-generic non-negative factors (red line) obtained by averaging the experimentally obtained profiles (gray lines) across subjects and conditions (speed × elevation)**. The non-negative factors have consistent shape and timing between the subjects and conditions.

The NNMF procedure resulted in four components being consistently extracted across all seven subjects and 25 locomotion conditions with a VAF of 94.1 ± 2% (mean ± standard deviation). Table 1 shows a summary of the timing of the peak activation, correlation and time shift of the extracted components across subjects and conditions. In this, the timing of the peak activation as well as the shape of the non-negative factors were consistent and repeatable across subjects and conditions. Consistency and repeatability is further highlighted in Figure 3, showing the average (red line) and standard deviation (gray shade) of the non-negative factors superimposed on all experimental factors.

**Table 1:** **Peak excitation, correlation and time shift of the extracted non-negative factors across conditions and subjects**.

**Component**	**Peak excitation (%)**	**Correlation (%)**	**Time shift (%)**
1	5.3 ± 3.2	95.2 ± 4.6	2.1 ± 2.2
2	37.9 ± 9.9	94.9 ± 3.6	3.5 ± 4.8
3	71.9 ± 2.4^*^ 97.9 ± 1.8^**^	89.9 ± 2.6	2.6 ± 6.6
4	91.3 ± 3.5	91.4 ± 5.6	1.7 ± 1.6

* Values for the first peak.

** *Values for the second peak. Refer to Figure 3*.

In component 1, the corresponding non-negative factors peaked in the transitions between heel strike and weight acceptance (5.3 ± 3.2% gait cycle, Table 1). Statistical tests showed significant weightings changes across speeds and elevations (*p* < 0.01) in muscles including: VastLat, VastMed, Rfem, Sar, GlutMed, Bfem, Semi, and GlutMax. The non-negative factors of component 2 peaked in the stance phase (37.9 ± 9.9% gait cycle, Table 1). Statistical tests showed significant weightings modulation across speeds and elevations (*p* < 0.01) in the Sol, Per, GastLat, and GastMed. In component 3 the non-negative factors exhibited a two-peak shaped profile (Figure 3). The first burst peak occurred approximately at 71.9 ± 2.4% of the gait cycle (i.e., after toe off), and the second one at 97.9 ± 1.8% (i.e., just before the heel strike). Statistical tests showed significant weightings modulation across speeds and elevations (*p* < 0.01) in the TA, Sar, and Add. Finally, component 4 marks the preparation for the heel strike at the end of the swing phase (peaked at 91.3 ± 3.5% of the gait cycle). The statistical test demonstrated significant differences across speeds and between elevations for the weightings of the TA, GlutMed, Semi, BFem (*p* < 0.01).

**Figure 4:**
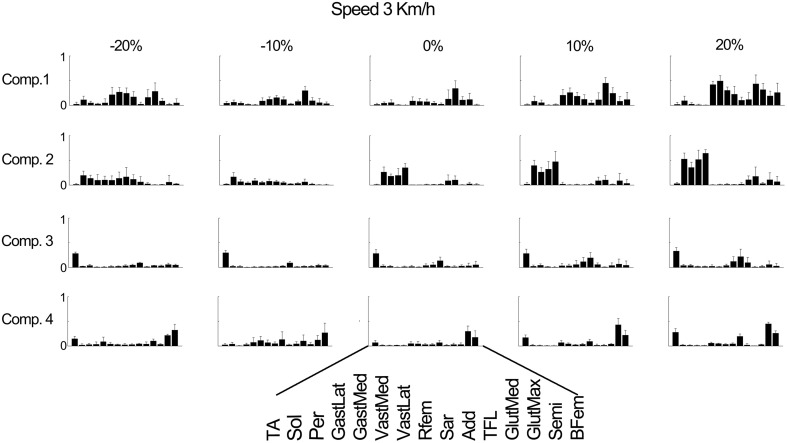
**Weighting coefficients for 15 muscles in four components at the speed of 3 km/h and across elevations**. The bars represent the average amplitude (mean ± standard deviation) for seven subjects.

**Figure 5:**
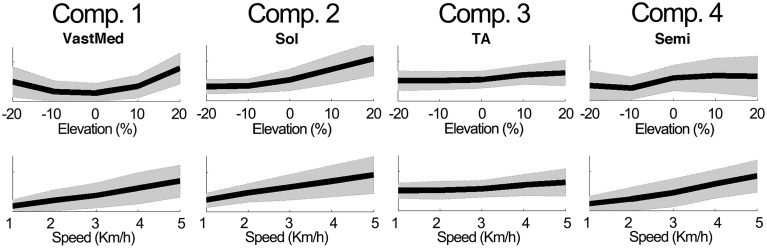
**Example modulations of muscle weightings across speeds and elevations**. The plots depict the mean ± standard deviation of the amplitude differences with respect to the baseline condition. For elevations (speeds), the mean was calculated by averaging across speeds (elevations).

Figure 4 further outlines the weightings for all muscles across all elevations for the speed of 3 km/h. For other speeds similar muscle weighting trends were obtained. Figure 5 illustrates the changes in muscle weightings for representative muscles in each component across four locomotion speeds and elevations. The figure depicts the difference in the weightings with respect to the values in the baseline condition (speed of 3 km/h and elevation 0%). Across speeds, the muscle weightings consistently increased with speed. The trend was the same for positive elevations, i.e., higher elevations were characterized with higher weightings, whereas the changes in muscle weightings for negative elevations were muscle-specific. These characteristic trends can be observed most clearly in the highly activated muscles of each component.

### Predictive modeling

#### Validation in scenario 1

**Figure 6:**
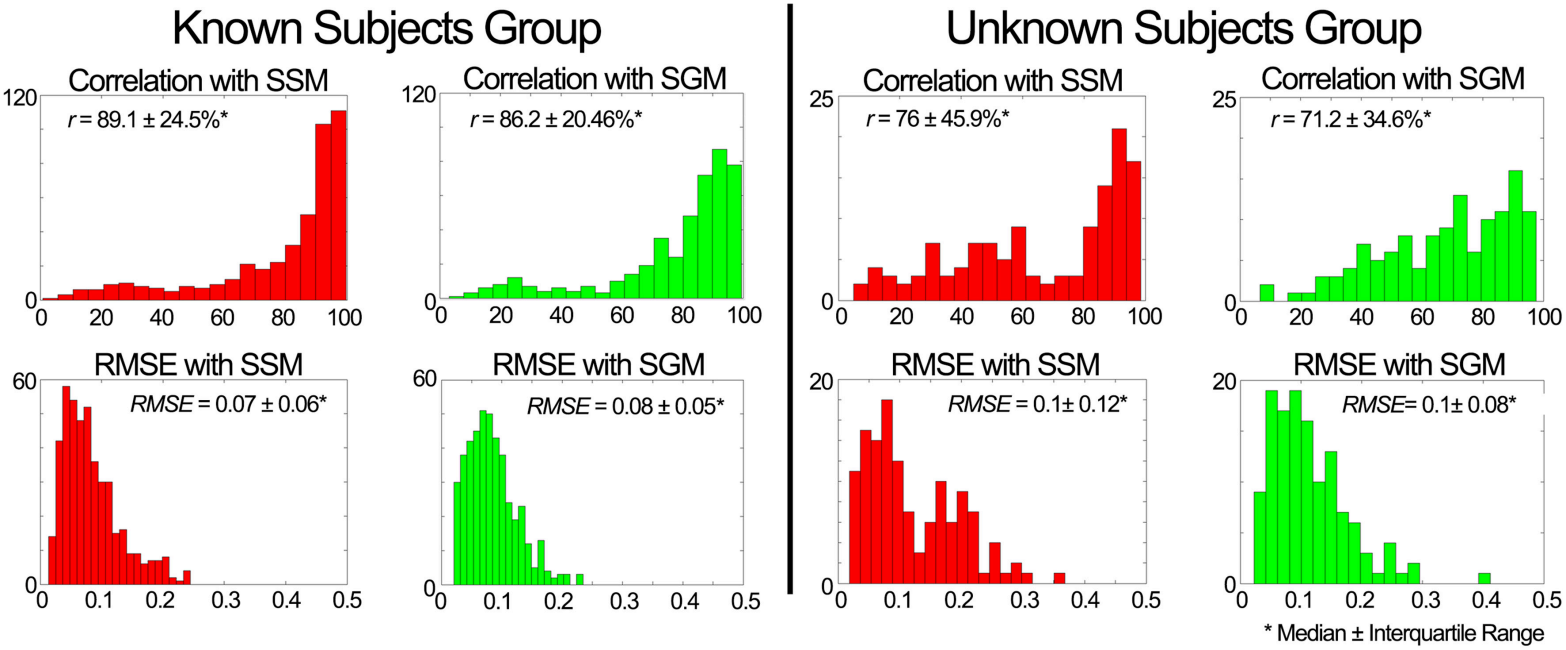
**Performance assessment of the weight predictor block in scenario 1**. The plots depict histograms of the average (median ± interquartile range) correlation coefficient (*r*) and root mean square error (RMSE) between the estimated and experimental weightings.

**Figure 7:**
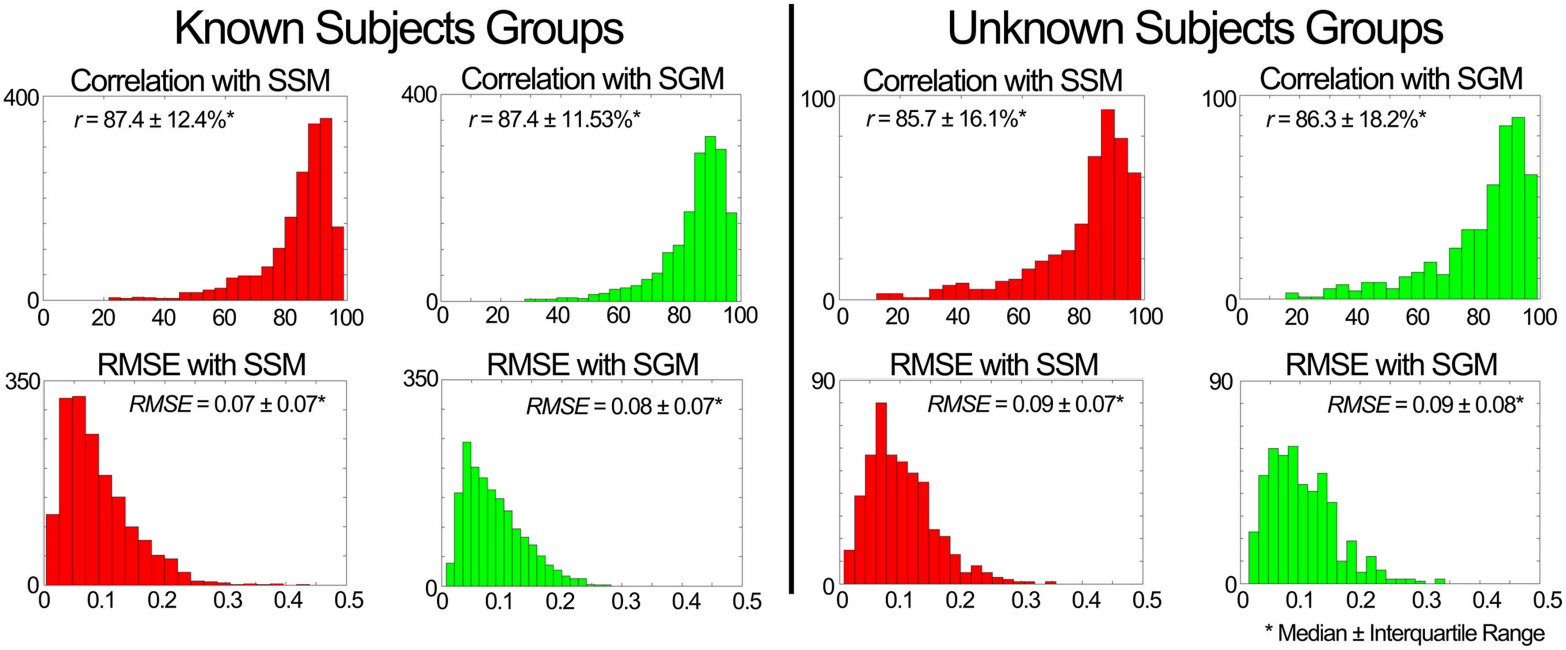
**Performance of the muscle excitation profiles (MEP) predictor block developed in scenario 1**. The plots depict histograms of the average (median ± interquartile range) correlation coefficient (*r*) and root mean square error (RMSE) between the estimated and experimental MEPs.

- **Evaluation of the XP block output**. Table 2 shows the correlation and time shift between the predictive model XPs and the experimental non-negative factors (Section Applicability of the developed MEP Model). It indicates that the XPs determined in this scenario correlated well with the experimental non-negative factors and closely approximated the maximum peak timings when tested in the unknown conditions for the known subjects group. Correlation values were always above 88% with time shifts below 9.5%.
  The results were similar for the unknown subject group (see Table 3). The correlation values were higher than 87% and the time shifts below 11% for most components and conditions. Therefore, although the model was trained using a restricted dataset (i.e., only 9 conditions, Section Descriptive Analysis), the resulting XP correctly approximates novel subjects. However, component 4 showed similar shape to experimental values (*r* >87%), but the peaks were considerably shifted as compared to other components, especially for elevation 10% (17.4 ± 11.1%) and both speeds (18.9 ± 6.3 and 17.7 ± 19.2%).
- **Evaluation of the weighting predictor block output**. Figure 6 summarizes the quality of estimation of the muscle weightings in unknown conditions for both the known and unknown groups using the two modes of the predictive model (SSM and SGM). For a total of 448 comparisons (i.e., seven subjects, four components, and 16 conditions) in the known subjects group, the SSM outperformed the SGM significantly (*p* < 0.01) in both outcome measures. However, for the unknown subject group, for a total of 128 comparisons (i.e., two subjects, four components, and 16 conditions) there was no statistically significant difference in the quality of estimation between the SGM and SSM. However, as seen in Figure 6 (right plots), the histograms show that SSM tends to be more skewed toward higher correlation in the estimation than SGM.
- **Evaluation of the MEP estimator block output**. Figure 7 shows a summary of results for the estimation of MEPs across unknown conditions. For both known and unknown subjects, the model in SSM and SGM estimated the MEPs with a median correlation higher than 85% and a median RMSE below 0.01 for novel conditions. For a total of 1680 comparisons (i.e., seven subjects, 15 muscles, and 16 condition) in the known subject group and 480 comparisons (i.e., two subjects, 15 muscles, and 16 conditions) in the unknown subject group, the performance was similar between modes, with no statistical differences found between them. This points out that the SGM captures well the average behavior of the experimental muscle excitations.

**Table 2:** Correlation and time shift between the predictive model XPs and the experimental non-negative factors for the known subjects group (mean ± standard deviation)^*^.

**Component**	**Correlation (*****r*****)**				**Time Shift (%)**			
	**Elevation**		**Speed**		**Elevation**		**Speed**	
	**−10%**	**10%**	**2 km/h**	**4 km/h**	**−10%**	**10%**	**2 km/h**	**4 km/h**
1	92.2 ± 4.2	95.8 ± 1.7	93.7 ± 4.5	94.4 ± 2.6	6.3 ±7.2	5 ± 6.1	5.3 ± 8.9	3.3 ± 2.4
2	95.3 ± 3.3	91.1 ± 5.1	93.3 ± 5.1	94.2 ± 3.8	7.15 ± 4.4	4.7 ± 3.9	6.6 ± 5.5	6.5 ± 9.9
3	89.3 ± 5.9	90.6 ± 6.5	92.8 ± 4.7	88.9 ± 7.4	6.95 ± 7.9	7.1 ± 4.6	5.2 ± 4.1	7.1 ± 5.1
4	90.4 ± 5.3	87.6 ± 6.1	90.8 ± 4.6	88 ± 6.7	6.19 ± 7.8	6.7 ± 6.6	9.5 ± 14.9	4 ± 2.7

* *For elevations, the average was computed across speeds and vice versa*.

**Table 3:** Correlation and time shift between the predictive model XPs and the experimental non-negative factors for the unknown subjects group (mean ± standard deviation)^*^.

**Component**	**Correlation (*****r*****)**				**Time Shift**			
	**Elevation**		**Speed**		**Elevation**		**Speed**	
	**−10%**	**10%**	**2 km/h**	**4 km/h**	**−10%**	**10%**	**2 km/h**	**4 km/h**
1	96.5 ± 2.4	96.2 ± 3	94.5 ± 7.5	96.6 ± 4.2	4.4 ± 2.9	4.7 ± 2.8	7.5 ± 11.4	10.2 ± 16.1
2	98.5 ± 0.5	93.5 ± 2.4	95.0 ± 3.4	95.7 ± 2	4 ± 2.4	5.6 ± 3.5	5.3 ± 3.8	6.8 ± 4.6
3	86.9 ± 7.1	91.4 ± 5.5	90.5 ± 4.5	90.9 ± 7.5	10.4 ± 11.6	5.4 ± 4.3	4.9 ± 5.2	4.8 ± 3.6
4	87.9 ± 9.7	93.9 ± 2.8	91 ± 7	87.6 ± 9.9	7.9 ± 5.6	17.4 ± 11.1	18.9 ± 6.3	17.7 ± 19.2

* *For elevations, the average was computed across speeds and vice versa*.

#### Validation in scenario 2

**Figure 8:**
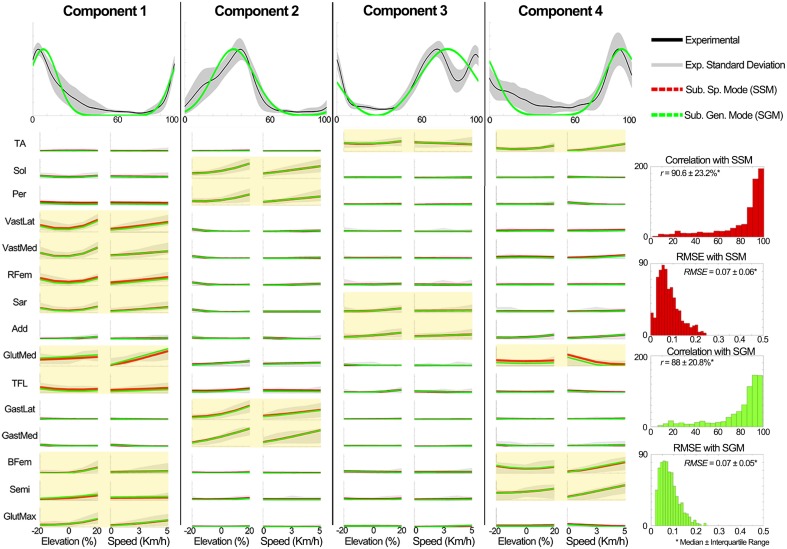
**Comparison of the generic XPs and the estimated the weightings in scenario 2 to the experimental data of the known subject group**. Experimentally obtained muscle modularity (non-negative factors and weightings) was well approximated by the predictive model. The estimation of weightings was better for highly active muscles in each component (yellow shaded plots). The y axes in all the plots are normalized between 0 and 1. The plots in the first row depict the XPs and the other plots show the weightings. In the plots for the elevations (speeds), the experimental weightings were averaged across speeds (elevations). For the GM and SM, the mean weightings are shown.

**Figure 9:**
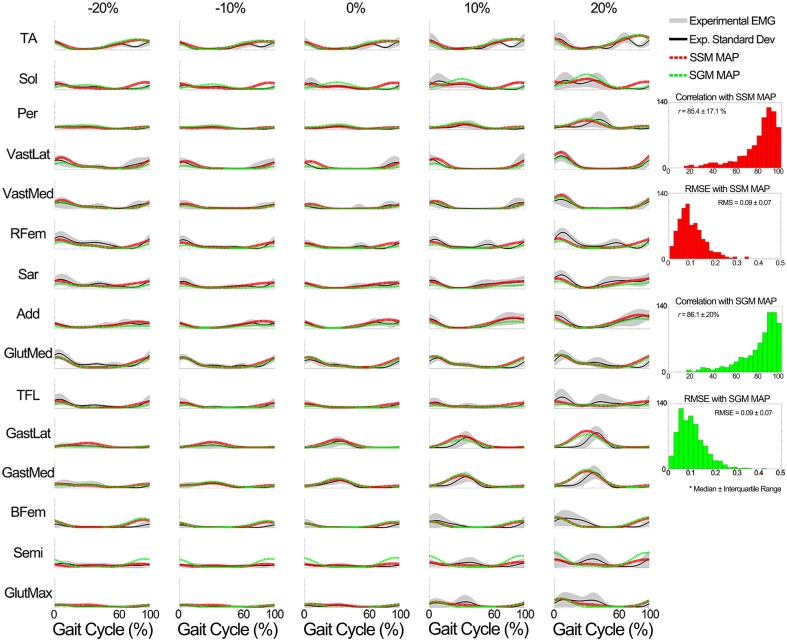
**Comparison of the output of the muscle excitation profiles (MEP) estimator block in scenario 2 to the experimental results for all tested muscles for the data of the unknown subjects group**. The results show that the estimated muscle excitation profiles closely approximated the experimental ones in both modes (the SM and GM). The y axes are normalized between 0 and 1.

- **Evaluation of the XP block output**. Table 4 shows that the XPs determined in scenario 2 approximated well the shape (*r* > 90%) and timing (time shift < 7.3%) of the experimental non-negative factors for both the known and unknown subject groups.
  **Evaluation of the weightings predictor block output**. Figure 8 shows a summary of the weighting prediction in scenario 2 for the known subject group. The yellow shaded areas highlight the predominantly recruited muscles in each component. Across a total of 700 comparisons (i.e., seven subjects, four components, and 25 conditions), the SSM showed a significant higher correlation and a significant lower RMSE (*p* < 0.01) than the SGM.
- The weightings predicted for the unknown subjects group correlated with the experimental weightings (median ± interquartile range) with *r* = 78.7 ± 45.7% for the SSM and 70.9 ± 32.6% for the SGM, and a RMSE of 0.08 ± 0.12 for the SSM and 0.10 ± 0.08 for the SGM, over a total of 200 comparisons (2 subjects, 4 components, 25 conditions).
- **Evaluation of the MEP estimator block output**. When compared to the experimental data of the known subjects group, MEPs were predicted with a correlation (median ± interquartile range) of ***r*** = 87.9 ± 12.3% for the SSM and ***r*** = 87.9 ± 12.1% for the SGM, over a total of 2625 cases (i.e., seven subjects, 15 muscles, 25 conditions), and the RMSE was 0.07 ± 0.07 for the SSM and 0.08 ± 0.06 for the SGM. No statistically significant differences were found between modes. Figure 9 shows a summary of the results obtained when the model was evaluated using the data from the unknown subjects group. In this group, the prediction was also similar with both modes (*r* > 85% and RMSE < 0.09), with no statistically significant differences between modes. Figure 9 shows a summary of the results obtained when the model was evaluated using the data from the unknown subjects group. In this group, the prediction was also similar with both modes (*r* > 85% and RMSE < 0.09), with no statistically significant differences between modes.

**Table 4:** **Correlation and time shift between the predictive model XPs and the experimentally obtained non-negative factors (mean ± standard deviation)**.

**Component**	**Known subjects**		**Unknown subjects**	
	**(7 subjects)**		**(2 subjects)**	
	***r* (%)**	**Time shift (%)**	***r* (%)**	**Time shift (%)**
1	93.9 ± 3.9	5.7 ± 7.9	96.4 ± 4.3	4.6 ± 2.8
2	93.4 ± 4.8	6.8 ± 8.3	98.4 ± 1.5	1.9 ± 1.1
3	90.1 ± 6.3	6.6 ± 5.3	93.2 ± 7.5	3.6 ± 3.0
4	91.1 ± 6.4	7.31 ± 9.8	93 ± 6.7	3.3 ± 5.9

## Discussion

In this study, we first employed a descriptive analysis to investigate how muscle modularity varied across 25 treadmill locomotion conditions including five speeds and five elevations across seven healthy individuals. We then created a predictive model that synthetized the observed regularities into a compact computational representation using Gaussian fitting and non-linear regression.

Current descriptive studies in the literature had never analyzed the modular structure of muscle excitations during locomotion across the large repertoire of conditions reported in this study. Importantly, our results showed that the average time shifts in the non-negative factors peaks were always less than 4% of the gait cycle across all conditions and subjects (Table 1). These results are consistent with what previously reported (Ivanenko et al., 2004) where time shifts in the extracted factor peaks were within 9% of the gait cycle in average. Based on these results Ivanenko and colleagues concluded that the extracted factors were robust during locomotion and that they were not highly dependent on locomotion conditions including: speed, step cycle duration and limb mechanical loading. In the light of these results, we modeled excitation primitives as Gaussian curves that preserve shape and timing across all considered elevations and speeds.

The muscle excitations were represented at the muscle-specific weightings level, which varied linearly or quadratically across conditions (Figures 4, 5). Our results were consistent, in terms of motor components and modularity, with previous descriptive work investigating muscle synergies during ground-level locomotion (Cheung et al., 2005; Ivanenko et al., 2006a; Neptune et al., 2009; Lacquaniti et al., 2012; Duysens et al., 2013; Walter et al., 2014) and across a subset of speeds (Ivanenko et al., 2004; Cappellini and Ivanenko, 2006).

The descriptive analysis provided a viable way to synthetize the observed modulations into a predictive model. The subject-invariant and condition-invariant non-negative factors were parameterized as a function of the gait cycle using Gaussian curves (Equation 1 and Figure 8). On the other hand, the variations observed in the discrete muscle weightings could be captured using a regression function (Equation 3). Results from scenario 1 (Section Predictive Modeling, Validation in Scenario 1, Figures 6, 7) highlighted the predictive model ability of estimating muscle modularity and excitations under novel locomotion conditions (elevations and speeds) and subjects (Figures 8, 9). Results also showed that the SGM well captured the features describing average muscle modularity and excitations across all subjects and locomotion conditions. On the other hand using the SSM enable higher accuracy on a trial-by-trial basis, since its estimations tend to be more skewed toward higher correlation values as seen in Figure 6. The main advantage of the SGM is that it operates as a pure function of speed and ground-elevation (i.e., without collecting any experimental EMG data), whereas the SSM model is customized to a specific subject by using experimentally collected EMG data from baseline locomotion. This is a minimal calibration dataset (only baseline condition is needed), which enables applicability to a range of input speeds and elevations.

Predicting muscle excitations using regression on time-varying signals involves the use of complex neural-networks and supervised machine learning methods, which have the disadvantages of (1) hiding the underlying modular structure of multi-muscle control and (2) further constraining the computational requirements needed for learning as well as its application. In this, the generalizability of the chosen machine learning method would decrease as a function of its complexity, i.e., number of neurons and connecting layers in a neural-network (Wang and Buchanan, 2002; Valero-Cuevas et al., 2009). Recent studies in the literature employed the theory behind muscle synergies to establish predictive models of muscle function (Rückert and d'Avella, 2013; Gopalakrishnan et al., 2014). The work in (Gopalakrishnan et al., 2014) aimed to provide a novel method for extracting muscle synergies from joint moment estimates. This was based on using musculoskeletal modeling and inverse dynamics to solve for the muscle excitation primitives and muscle weightings required to track target joint moments during locomotion tasks at two different speeds. It is worth stressing that the study made the assumption that muscle weightings were expected to be invariant across locomotion speeds while excitation primitives were expected to vary. This is in contradiction with respect to what our descriptive analysis found (See Section Descriptive Analysis) as well as with respect to previous descriptive work of muscle synergies across different locomotion conditions, which state that excitation primitives are considered to be invariant across locomotion speeds, while muscle weightings undergo locomotion speed-related modulation (Hansen et al., 2004; Ivanenko et al., 2004, 2006b; Cappellini et al., 2010). The work in (Rückert and d'Avella, 2013) aimed to provide a theoretical and computational framework that exploited similarities and shared synergies across different motor tasks to enable robust motor skill learning in multi-body dynamic systems. This was done by using superposition of a set of basis functions for determining movement trajectories in the considered dynamic models. These previous studies directly employed dynamic simulation of the human musculoskeletal system, thus making them comparable to our earlier work on synergy-driven musculoskeletal modeling (Sartori et al., 2013) as well as to other related work (McGowan et al., 2010; Allen and Neptune, 2012; Walter et al., 2014).

The work we now propose does not employ musculoskeletal modeling. It first employs a descriptive analysis for identifying the muscle synergies underlying experimental lower extremity electromyograms. Afterwards, it synthetizes the experimentally observed synergies into a predictive model using Gaussian-fitting and non-linear regression. Our proposed descriptive analysis and predictive model encompass a larger repertoire of locomotion conditions than what has been reported in literature. We argue that our proposed approach will be central for informing muscle excitation-driven musculoskeletal simulations of a large repertoire of human locomotion conditions (also see Section Applicability of the developed MEP Model), a scientific area where the theory of muscle synergy is increasingly gaining importance (McGowan et al., 2010; Allen and Neptune, 2012; Rückert and d'Avella, 2013; Sartori et al., 2013; Gopalakrishnan et al., 2014; Walter et al., 2014).

Finally, our proposed model has the advantage from similar approaches that it is synthesized from experimental data and parameterizes an extensive set of continuous muscle excitations using two discrete numbers (desired speed and elevation) and a set of baseline weightings. This was possible since the set of generic XPs encoded the temporal modulation shared by all the muscles, while the weightings encode the muscle-specific excitation amplitude across the conditions. As a result, our proposed predictive model is convenient for implementation, even in systems with limited computational resources (e.g., embedded devices). The system only needs to store a set of parameters (expression coefficients) and execute basic scalar and matrix operations. To the best of our knowledge, there is no other model characterized with the similar flexibility, which accommodates many muscles and conditions. In addition, it explicitly preserves the modular structure underlying the observed muscle activation profiles. This is important since both the synthesized XPs and the estimated weightings are of interest for potential applications, as discussed in the next section.

### Applicability of the developed mep model

Our proposed predictive model offers many advantages in different applications domains. We will outline 4 specific applications that will be the objective of future research:

- **Functional electrical stimulation (FES) controller:** The proposed predictive model can be used to implement control strategies in neuroprostheses for restoring motor functions in patients with neurological conditions. It can provide MEPs across a large variety of locomotion conditions, which can serve as the templates for designing stimulation profiles of an FES system on targeted muscles during the gait cycle. This is an important aspect as the main challenge behind developing FES-controllers is the inability of determining desired neuromuscular excitation patterns for a large number of muscles across a wide range of locomotion conditions. There are studies in literature in which stimulation profiles were designed by mimicking experimentally recorded EMGs, but they considered only a limited set of muscles and conditions (e.g., single muscle O'Keeffe et al., 2003, speed modulation Byrne et al., 2007). The model presented here would enable this approach to be generalized to multi-muscle systems, in which the stimulation profiles could be updated online based on the parameters (desired speed and elevation) supplied by the higher levels of control. Also, by exploiting the concept of muscle modularity, the model could be used as an event-based guideline to deliver the stimulation at the correct time to only relevant muscles as discussed by Piazza et al. (2012).
- **Assessment and biofeedback:** The estimated muscle modularity (XPs and weightings) could be used as a healthy benchmark to guide and evaluate the rehabilitation of human locomotion (Galeano et al., 2014). Due to the low computational requirements, these procedures could be implemented online (e.g., during rehabilitation). For example, during treadmill or robotic training the current walking speed and elevation can be measured using kinematic sensors and the predictive model can provide reference muscle synergies characterizing healthy walking in the current condition. The reference can be compared to the actual synergies extracted online from the patient muscle activity in order to assess the recovery and/or provide biofeedback to the patient, facilitating the convergence toward a healthy pattern.
- **Modeling:** In the context of movement analysis, neuromusculoskeletal modeling has been widely used to understand how neuromuscular control contributes to produce dynamic musculoskeletal movement (Zajac et al., 2002; Pandy and Andriacchi, 2010; Fregly et al., 2012). In this scenario, surface EMG envelopes have been used to directly drive individual musculotendon units in subject-specific models that can predict dynamically consistent joint moments, forces, and motions (Lloyd and Besier, 2003; Buchanan et al., 2004; Barrett et al., 2007; Sartori et al., 2012; Gerus et al., 2013). This however necessitates the availability of experimental EMG data. Alternatively, optimization has been used as a way to solve for the redundancy in the musculoskeletal system (Anderson and Pandy, 2001; Erdemir et al., 2007; Seth and Pandy, 2007). However, these methods rely on pre-defined optimization criteria that do not necessarily generalize across locomotion conditions. Furthermore, static optimization-based methods are currently unable to predict neuromuscular mechanisms including physiological muscle pre-activation and co-activation ratios (Tax et al., 1990; De Serres and Milner, 1991; Buchanan and Lloyd, 1995; Norton and Gorassini, 2006; Menegaldo and Oliveira, 2011). In this context, our proposed model can provide an initial pattern of synthetic muscle excitations that well describe the electrophysiology underlying the condition-specific locomotion. These patterns can be used as an initial feedforward solution to inform hybrid musculoskeletal simulations. These reproduce the musculoskeletal dynamics underlying a given motor task by minimally adjusting the initial feedforward muscle excitations (Sartori et al., 2014). This will enable generating dynamic musculoskeletal simulations of locomotion that are consistent both dynamically (i.e., match experimental joint dynamics) and electrophysiologically (i.e., match experimental EMG data) with no need for recording experimental EMG signals (predictive model in SGM) or with minimal experimental EMG (predictive model in SSM).
- **Biped robotic control:** The proposed model can be used to provide further solutions for synthetizing human-like locomotion in simulation or in artificial bipedal systems (Degallier and Ijspeert, 2010). In this context, the predictive model can generate prototype patterns of the human-like feedforward motor commands in muscle space (predictive model) or joint actuator space (predictive model informing the musculoskeletal simulation), which can be used for designing a biologically inspired control. The predictive model could serve as an efferent component within the overall control structure, which also implements afferent loops. Furthermore, the XPs provide level-of-recruitment profiles which are impulsive and timed relative to the gait cycle and/or events and are thereby similar to the central pattern generator components in the human motor control and robotics (Ijspeert, 2008; Degallier and Ijspeert, 2010). Certainly, all the above-mentioned scenarios require future research work and systematic validation.

## Conclusions

The primary contributions of our study are that (1) it characterized how muscle modularity varies across a large spectrum of locomotion conditions (5 speeds and 5 elevations), and (2) used the observed modular structure to design a predictive framework that was validated across novel conditions and individuals. The developed predictive model is computationally efficient and therefore convenient for real-time operation. It can provide muscle-specific excitations as well as the modular structure (excitation primitives and weightings) underlying the desired locomotion condition. The results showed that the model could be used in two different modes to predict a large repertoire of excitation for up to 15 muscles for one leg during locomotion in different elevations and speeds. We developed a subject-generic mode, which does not need collecting experimental data, and a subject-specific mode, which only needs collecting data for a baseline condition. This allowed us to synthesized the neuromuscular mechanisms underlying locomotion with important implications in neurorehabilitation technologies. Open-access of the model implementation is provided for further analysis at https://simtk.org/home/p-mep/.

## Conflict of interest statement

The authors declare that the research was conducted in the absence of any commercial or financial relationships that could be construed as a potential conflict of interest.

## Footnotes

^1^http://www.seniam.org/
