# Supplementary material for: A predictive model of muscle excitations based on muscle modularity for a large repertoire of human locomotion conditions
Source: Front Comput Neurosci. 2015 Sep 17;9:114. doi: 10.3389/fncom.2015.00114 (PMC4585276; doi:10.3389/fncom.2015.00114)
Supplement: Supplementary file 1 [file ModelDevelopment.PDF]

## *Supplementary Material*

# A compact predictive model of muscle modularity during locomotion across elevations and speeds

**Jose Gonzalez-Vargas [1]*, Massimo Sartori [2], Strahinja Dosen[2], Diego Torricelli[1], Jose L. Pons[1], Dario Farina[2]**

[1]Neural Rehabilitation group, Cajal Institute, Spanish National Research Council, Madrid, Spain
[2]Department of Neurorehabilitation, University Medical Göttingen, Göttingen, Germany

*** Correspondence:** Corresponding Author, Neural Rehabilitation group, Cajal Institute, Spanish National Research Council, Madrid, Spain, je.gonzalez@csic.es

## 1. Development of Predictive Model

**1.1. Determining excitation primitives (Fig. 1A).** Descriptive analysis results revealed that the subject-specific and condition-specific non-negative factors for a given motor component were highly similar and repeatable in terms of shape and timing (see Methods and Results in Sections 3.1 and 4.1). All factors for a specific motor component were therefore averaged across subjects and conditions and normalized to the overall maximum value yielding a subject-generic and condition-generic factor for that motor component. These profiles were then fitted by Gaussian curves using non-linear least-square method (Sartori et al., 2013). The result comprised four components that can be explained by Eq. 1.

$$\widetilde{XP}_c(t) = e^{-\frac{(t-\mu_C)^2}{2\sigma_C{}^2}}, \tag{1}$$

where $t$ is the gait cycle frame (i.e. 0% ≤ t ≤ 100% gait cycle), $\mu_C$ is the temporal shift of the peak of the Gaussian curve within the gait cycle, and $\sigma_C$ is the width of the Gaussian curve for each extracted component ($c$). This step was performed only once as it captures XPs descriptive of all subjects and locomotion conditions. The final resulting XPs were used for all tests made in this study.

**1.2. Calculating speed- and velocity-dependent weighting increments (Fig 1B).** In order to develop a regression model to predict the muscle weightings, we generated a dataset to use for training the model. For this purpose we calculated the differences in the weightings of all locomotion conditions with respect to a baseline condition (i.e. speed = 3km/h and elevation = 0%) for each subject. Specifically, the speed-dependent differences ($\Delta v$), for a specific muscle ($m$) within a specific component ($c$), were calculated as increments with respect to the weightings at a baseline speed ($i_{BL}$ = 3) for all elevations ($j$ = *-20, -10, 0, 10, 20* %) and subjects ($s$), as described in Eq.2:

$$\Delta v_{i,j,m,c,s} = y_{i,j,m,c,s} - y_{i_{BL},j,m,c,s} \tag{2}$$

where $y$ is the experimental muscle weighting and $i$ = *1,2,3,4,5* km/h. The baseline speed was chosen to be 3 km/h because it is close to the normative walking speed and therefore easily reproducible across experiments and individuals.

Analogously, the elevation-dependent increments ($\Delta\theta$) in the muscle weightings, for a specific muscle ($m$) within a specific component ($c$), were computed as increments with respect to the weightings at a baseline elevation ( $j_{BL}$ = 0) for all speeds ($i$ = *1,2,3,4,5* km/h) and subjects ($s$), as described by Eq. 3.

$$\Delta\theta_{i,j,m,c,s} = y_{i,j,m,c,s} - y_{i,j_{BL},m,c,s} \tag{3}$$

where $y$ is the experimental weighting and $j$ = *-20, -10, 0, 10, 20*%. The baseline elevation ($j_{BL}$) was chosen to be 0% ($j_{BL}$ = 0) by using the same rational as before (normative condition).

**1.3. Determining a regression model for the increments (Fig. 1C).** Across all subjects and conditions the experimental speed- and elevation-weighting increments calculated in the previous step, exhibited quasi-linear or quadratic trends as determined by a descriptive analysis (see section 4.1). Therefore, quadratic equations were selected as the regression models. The final regression model was comprised of two equations per component ($c$) and per muscle ($m$), one (Eq. 4) for speed-dependent and the other (eq. 5) for elevation-dependent increments:

$$\Delta v_{m,c}(v) = a_{m,c}v^2 + b_{m,c}v + d_{m,c} \tag{4}$$

$$\Delta\theta_{m,c}(\theta) = A_{m,c}\theta^2 + B_{m,c}\theta + D_{m,c} \tag{5}$$

where the parameters *a, b,* and *d* correspond to the fitted coefficients of the quadratic equation characterizing the speed-dependent changes, while the parameters A*, B,* and *D* characterize the elevation-dependent changes of the increments.

**1.4. Correction of the weightings predictor block (Fig. 1E).** The weightings predictor block estimates the muscle weightings by adding the predictive speed-dependant increments (Eq. 4), the elevation-dependant increments (Eq. 5), and the baseline ($W_{BL_{m,c}}$,) This prediction is described by Eq. 6.

$$W_{m,c}(v,\theta,W_{BL}) = \Delta v(v)_{m,c} + \Delta\theta(\theta)_{m,c} + W_{BL_{m,c}} \tag{6}$$

Finally a correction term is used to remove the offset introduced by the regressions model at baselines values (Eq. 7). This guaranties that the estimated muscle weightings values are equal to experimental ones at the baseline. Also, the correction assures that the estimated muscle weighting values are non-negative.

$$\widetilde{W}_{m,c}(v,\theta,W_{BL}) = \begin{cases} W_{m,c}(v,\theta,W_{BL}) - W_{m,c}(v_{BL},\theta_{BL},W_{BL}), & if\ \widetilde{W}_{m,c}(v,\theta,W_{BL}) \geq 0 \\ 0, & if\ \widetilde{W}_{m,c}(v,\theta,W_{BL}) < 0 \end{cases} \tag{7}$$
